# Intestinal parasites infecting captive non-human primates in Italy

**DOI:** 10.3389/fvets.2023.1270202

**Published:** 2024-01-08

**Authors:** Silvia Rondón, Serena Cavallero, Margherita Montalbano Di Filippo, Claudio De Liberato, Federica Berrilli, Nazareno Capitani, Stefano D’Amelio

**Affiliations:** ^1^Department of Public Health and Infectious Diseases, Sapienza University of Rome, Rome, Italy; ^2^Department of Food Safety, Nutrition and Veterinary Public Health, Istituto Superiore di Sanità, Rome, Italy; ^3^Istituto Zooprofilattico Sperimentale del Lazio e della Toscana “Mariano Aleandri”, Rome, Italy; ^4^Department of Clinical Sciences and Translational Medicine, University of Rome Tor Vergata, Rome, Italy; ^5^Parco Faunistico Piano dell’Abatino, Rieti, Italy

**Keywords:** captive primates, intestinal parasites, Italy, molecular characterization, zoonosis

## Abstract

Non-human primates (NHPs) living in captive conditions are susceptible to intestinal parasites that can contribute to mortality and morbidity, and cause zoonotic infections. Thus, parasite surveys on NHP populations under human care are relevant as part of the evaluation of NHPs welfare and in the zoonotic disease risk assessment, as well as in the exploration of parasite transmission pathways, according to the One-Health concept. This study aimed to identify intestinal parasites infecting NHPs living in two wildlife recovery centers and in a zoological garden, in Italy. Ninety-three fecal samples from *Macaca tonkeana, Macaca fascicularis, Sapajus apella, Chlorocebus aethiops, Macaca fuscata, Macaca sylvanus,* and *Cebus capucinus* were collected at Piano dell’Abatino Park (Lazio), and fecal smears and flotation were performed in order to identify parasites according to morphological keys. Additionally, one carcass of *M. fuscata* from the Bioparco Zoological Garden of Rome (Lazio) and one of *M. fascicularis* from the Center for the Recovery of Exotic and Maremma Wild Animals (Tuscany) were necropsied and intestinal adult nematodes were collected and characterized at morphological and molecular level, using the mitochondrial cox1 and rrnL markers. Protozoans (*Entamoeba coli, Iodamoeba bütschlii, Dientamoeba fragilis*-like, *Giardia* sp.), chromists (*Balantidium/Buxtonella* sp.) and nematodes (*Capillaria* sp., *Trichuris* sp., strongyliform larvae and *Oesophagostomum* sp.) were found through fecal smears and flotation. The collected adult nematodes from dead NHPs were morphologically identified as whipworms (genus *Trichuris*). Phylogenetic analyses grouped *Trichuris* specimens into the *Trichuris trichiura* complex of species, with specimens from *M. fuscata* clustering into a host-specific branch, and whipworms from *M. fascicularis* clustering within a clade formed by *Trichuris* infecting several primate species, including humans. The results here collected revealed the presence of potentially zoonotic parasites circulating in captive primates in Italy, providing useful information for the formulation of management and care plans for captive NHPs, and for the elaboration of safety measures for visitors and animal keepers.

## 1 Introduction

Intestinal parasites are often responsible for diseases in animals living in confined environments such as sanctuaries, zoological gardens and wildlife rescue centers (1). Captive animals may be more susceptible to protozoan and helminth parasites with direct life cycles, which are more prevalent and prone to disseminate in confined conditions where the animals might be more stressed due to overpopulation and malnutrition, showing clinical signs as diarrhea and dehydration, and requiring veterinary care (2, 3). Parasite transmission mainly occurs through the fecal-oral route via direct contact with infected hosts (or their fecal material), or indirectly through the ingestion of contaminated water or food (4). In a confined environment, the low hygienic measures may lead to high levels of environmental contamination, and the handlers’ movements among different premises without safe and hygiene measures may contribute to the dissemination of such parasites inside and outside the workplace. Captive non-human primates (NHPs) may act as reservoirs for zoonotic parasites and the frequent use of pharmacological treatments may lead to the selection of resistance traits (1, 5). Therefore, confined environments are of great interest for parasitological studies, involving the One-Health concept.

Parasitological investigations have been carried out worldwide in zoological parks housing NHPs. For instance, *Giardia duodenalis* infections were reported in several NHPs hosted in 12 zoological gardens in China (6), while in a study carried out in Malaysia in three zoos hosting 69 specimens of NHPs, there were reported 21 species of intestinal parasites with a high prevalence of nematodes like *Ascaris* spp. and *Oesophagostomum* spp., only one animal positive to *Blastocystis* and no observation of *Giardia* spp. (7). Moreover, a large survey on intestinal parasites infecting NHPs hosted in two research centers in Brazil reported a large occurrence of *Balantidium coli* and *Entamoeba* sp. among protozoans, and a general low frequency of helminths, with predominance of *Trichuris trichiura* (8).

In Europe, parasitological surveys on NHPs have been performed in zoological enclosures such as the Dublin Zoological Garden (Ireland) (9), the Belgrade Zoo (Serbia) (10), the Kiev Zoo (Ukraine) (11), the Brno Zoological Garden (Czech Republic) (12), the Sofia Zoo (Bulgaria) (13), the Wroclaw Zoo (Poland) (14), the Košice Zoological Garden (Slovakia) (15), among others (13, 16). Nematodes (e.g., *Ascaris* sp., *Trichuris* sp., *Strongyloides* sp.) are the most common parasites detected, followed by cestodes and trematodes (13). Furthermore, *G. duodenalis*, *Cryptosporidium hominis, Blastocystis* sp., and *Entamoeba dispar* circulation between NHPs and their zookeepers has been identified in European zoological gardens, with the confirmation of zoonotic transmission events involving *Blastocystis* sp. and a highly suspected zoonotic transmission of *C. hominis* (4). Additionally, subcutaneous *Taenia crassiceps* cysticercosis in a ring-tailed lemur in a Serbian zoo has been reported (17).

In Italy, some surveys on intestinal parasites infecting NHPs living in zoological gardens have been conducted so far. In central Italy, *Cryptosporidium* sp. and *Trichuris* sp. have been found infecting *Lemur catta* at the Giardino Zoologico of Pistoia (18), while, at the Bioparco Zoological Garden of Rome, *G. duodenalis* has been reported infecting *L. catta,* and *Entamoeba* spp. was diagnosed in *Cercocebus torquatus*, *Chlorocebus aethiops*, *Macaca fuscata*, *Mandrillus sphinx*, *Pan troglodytes*, *L. catta*, and *Pongo pygmaeus* (19). In southern Italy, *Trichuris* sp., *Strongyloides fuelleborni*, and *Cryptosporidium* sp. infected *Papio cynocephalus* at the Fasano Zoo Safari, while *G. duodenalis* was found infecting *L. catta*, *Cercopithecus mona*, *Alouatta caraya*, *Nomascus concolor*, *Colobus guereza*, and *Semnopithecus entellus* in a zoological garden in the Benevento province (20). Moreover, *Cyclospora* was detected in *P. troglodytes* from a wildlife animal rescue center, and in *Macaca fascicularis* from an experimental primate research center (21). Eight taxa of intestinal parasites (*Trichuris* sp., *Oesophagostomum* sp., *Entamoeba coli*, *Endolimax nana*, *Iodamoeba bütschlii*, *Chilomastix mesnili*, *B. coli*, and *Blastocystis* sp.) were recorded infecting *M. fascicularis* in a biomedical research center (22).

Concerning necropsies carried out on dead captive NHPs, *Trichuris* sp. from *Eulemur albifrons*, and *Strongyloides* sp. from *Macaca sylvanus* have been found at the Natura Viva zoo (23), and *Echinococcus granulosus* from *L. catta* at a zoo in northern Italy (24). At the Bioparco Zoological Garden of Rome, larval forms of *Taenia martis* from *L. catta* (25), and adult *Trichuris* sp. from *L. catta*, *M. fuscata* and *C. aethiops* have been reported (26, 27). Larval forms of *Mesocestoides* sp. from *Saguinus midas* were collected at a wildlife recovery center (28).

Despite their importance in public health and NHPs welfare, the currently available information on intestinal parasites infecting captive NHPs in Italy is still limited to fragmented data. Thus, here we provide a survey on intestinal parasites circulating in NHPs hosted in two wildlife rescue centers and in one zoological garden in central Italy.

## 2 Materials and methods

Fecal samples and adult nematodes were collected during 2020–2022, from NHPs living in confined environments in Italy.

### 2.1 Fecal samples

Ninety-three fecal samples from *Macaca tonkeana* (Tonkean black macaque) (*n* = 23), *Macaca fascicularis* (long-tailed macaque) (*n* = 16), *Sapajus apella* (tufted capuchin) (*n* = 43), *Macaca fuscata* (Japanese macaque) (*n* = 2), *Macaca sylvanus* (Barbary macaque) (*n* = 4), *Chlorocebus aethiops* (grivet) (*n* = 2) and *Cebus capucinus* (capuchin monkey) (*n* = 3) were collected at the Piano dell’Abatino Park (Lazio), in the framework of a routine parasitological survey. In this habitat, the animals are hosted in different premises, as detailed below. One premise hosts two individuals of *C. aethiops* and two individuals of *C. capucinus*; one premise is dedicated to *M. fascicularis*, with seven individuals; two not-separated premises for *M. sylvanus* with 11 individuals; one premise for only one individual of *M. fuscata*; three premises for *S. apella*, with 11, 12, and 14 individuals; and four premises for *M. tonkeana* with eight, six, thirteen, and fourteen individuals. Fresh samples were collected directly from the soil inside the premises and were not attributed to a specific individual. For each sample, one aliquot was stored in 10% formalin solution, and one aliquot in 70% ethanol solution. Samples were examined both macroscopically, to verify the presence of nematodes or cestodes, and microscopically. Morphological identification of protozoan and helminth parasites was performed after direct fecal smears (29) and flotation with a salt-sugar solution (SG: 1.28) (30) useful for general purposes. Slides from direct fecal smears and flotation were examined with a microscope, and at least 10 fields were screened at objective magnification ×100, ×200, ×400, and ×1,000, successively. This protocol was used to qualitatively identify parasite eggs, cysts and oocysts. Photos of parasites were taken for morphological identification. For some parasite taxa the identification was possible only to the genus level.

### 2.2 Adult nematodes

Two dead macaques were inspected during necropsies carried out at the Istituto Zooprofilattico Sperimentale del Lazio e della Toscana “Mariano Aleandri” to identify the cause of death. Ten entire adult nematodes (three males and seven females) and few disrupted nematode body portions were collected from the caecum of one *M. fascicularis* hosted at the Center for the Recovery of Exotic and Maremma Wild Animals (CREMWA) (Tuscany). From one *M. fuscata* hosted at the Bioparco Zoological Garden of Rome (Lazio), eight adult nematodes (all females - not well preserved) were collected from the caecum. Nematodes were repeatedly washed with saline solution, and then used for morphological observation after clarification in lactophenol. A body portion was used for molecular characterization based on sequence analyses of the two partial mitochondrial regions *cox*1 and *rrn*L, informative for phylogenetic assignment (31, 32). The obtained sequences were compared to homologous GenBank retrieved data, and used for phylogenetic inferences with the maximum likelihood (ML) method by MEGA7 (33), after testing for the best evolutionary models explaining the data (33). The only available homologous sequences of *Trichuris* sp. from the same host species *M. fascicularis* (JF690967) was not reliably attributable to this genus, thus it was excluded from the analysis. Sequences of *Trichinella spiralis* and *Trichinella britovi* were used as outgroups (AF293969, KM357413). Additional file 1 and file 2 show the material used for comparative analyses.

## 3 Results

### 3.1 Fecal samples

**Figure 1:**
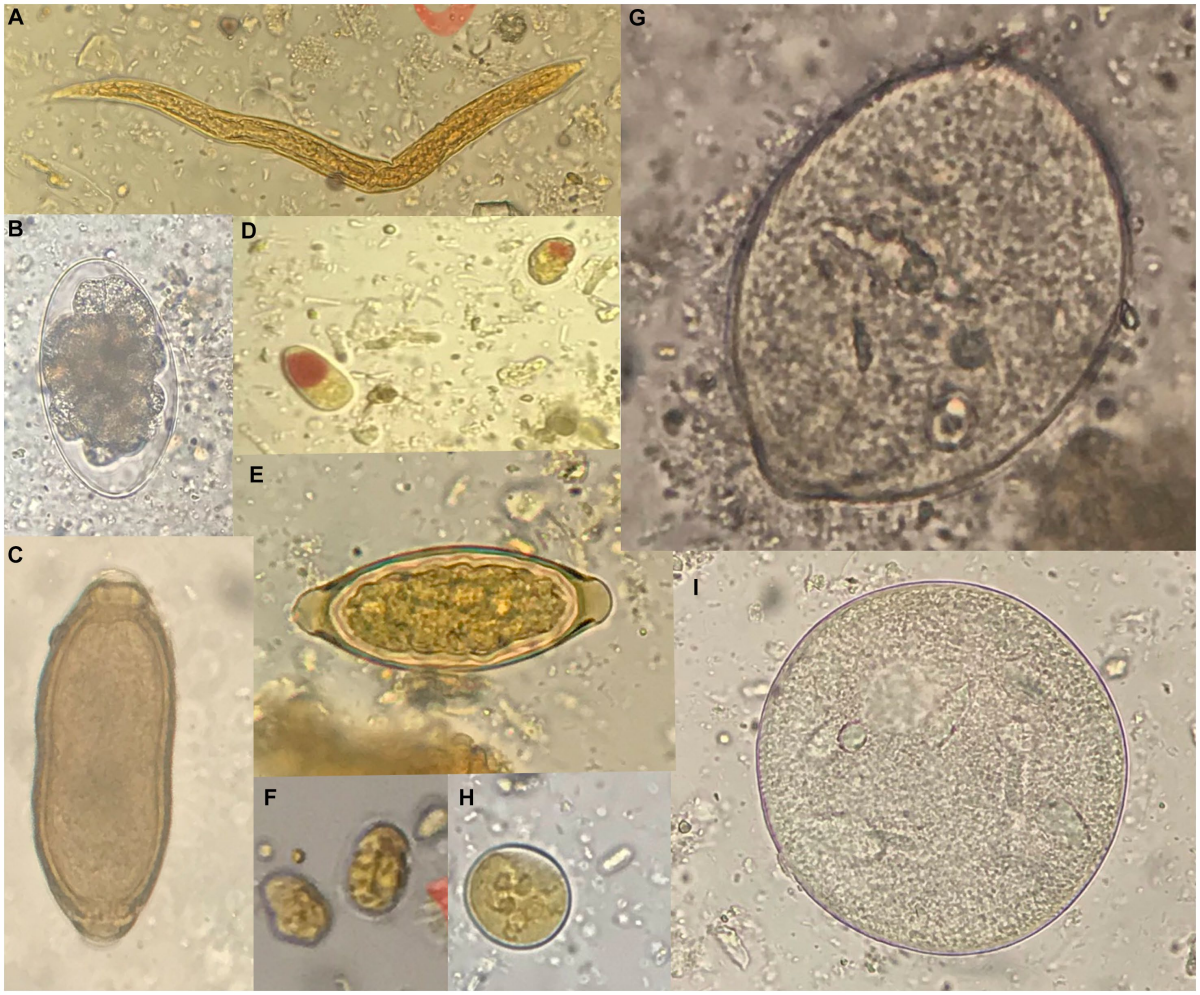
Representative images of parasites detected by microscopy. **(A)** Strongyliform larva (40x). **(B)** *Oesophagostomum* sp. (50 × 85 μm). **(C)** *Capillaria* sp. (40 × 25 μm). **(D)** *Iodamoeba bütschlii* (10 × 12 μm). **(E)** *Trichuris* sp. (25 × 55 μm). **(F)** *Giardia* sp. (10 × 8 μm). **(G)** *Balantidium/Buxtonella* sp. trophozoite (90 μm). **(H)** *Entamoeba coli* (20 μm). **(I)** *Balantidium/Buxtonella* sp. cyst. Measures refer to the samples shown in the figure.

Four taxa of protozoans (*Entamoeba coli, Iodamoeba bütschlii, Dientamoeba fragilis*-like, and *Giardia* sp.), one taxon of chromist (*Balantidium/Buxtonella* sp.), and four taxa of helminths (*Capillaria* sp., *Trichuris* sp., strongyliform larvae and *Oesophagostomum* sp.) were identified in the fecal samples from NHPs living at the Piano dell’Abatino Park (Table 1). None infected animals showed gastrointestinal symptoms. Representative images from microscopic analyses are available in the Figure 1.

**Table 1:** List of parasites identified according to host species.

**Parasite taxa**	***Cebus capucinus*** (*n* = 3 *N* = 2)	***Chlorocebus aethiops*** (*n* = 2 *N* = 2)	***Macaca fascicularis*** (*n* = 16 *N* = 7)	***Macaca fuscata*** (*n* = 2 *N* = 1)	***Macaca sylvanus*** (*n* = 4 *N* = 11)	***Macaca tonkeana*** (*n* = 23 *N* = 41)	***Sapajus apella*** (*n* = 43 *N* = 37)
*Entamoeba coli*	0	0	10	2	4	23	0
*Iodamoeba bütschlii*	0	2	8	1	1	11	4
*Giardia* sp.	0	1	0	0	0	0	0
*Dientamoeba fragilis-*like*	0	0	6	0	0	0	0
*Balantidium/Buxtonella* sp.	0	0	7	2	1	17	1
*Capillaria* sp.	0	0	0	0	0	0	1
*Trichuris* sp.	0	0	4	0	0	0	0
Strongyliform larvae*	0	0	0	0	1	0	5
*Oesophagostomum* sp.	0	0	0	0	0	1	0

Number of positive samples for each parasite taxa, per primate species (*n*, number of samples analyzed; N, number of primates hosted in the premises). *Based on morphology it was not possible to identify the genus/species.

The capuchin monkeys were the only primate species in which no gastrointestinal parasites were observed. The following potentially zoonotic parasites were detected: *Giardia* sp. was found infecting the grivet, *Trichuris* sp. infecting the long-tailed macaque, *Oesophagostomum* sp. was observed in the Tonkean black macaque and *Capillaria* sp. in the tufted capuchin monkey. *Trichuris* sp. and *Capillaria* sp. were not identified at species level due to negative results of molecular identification assays.

### 3.2 Adult nematodes

**Figure 2:**
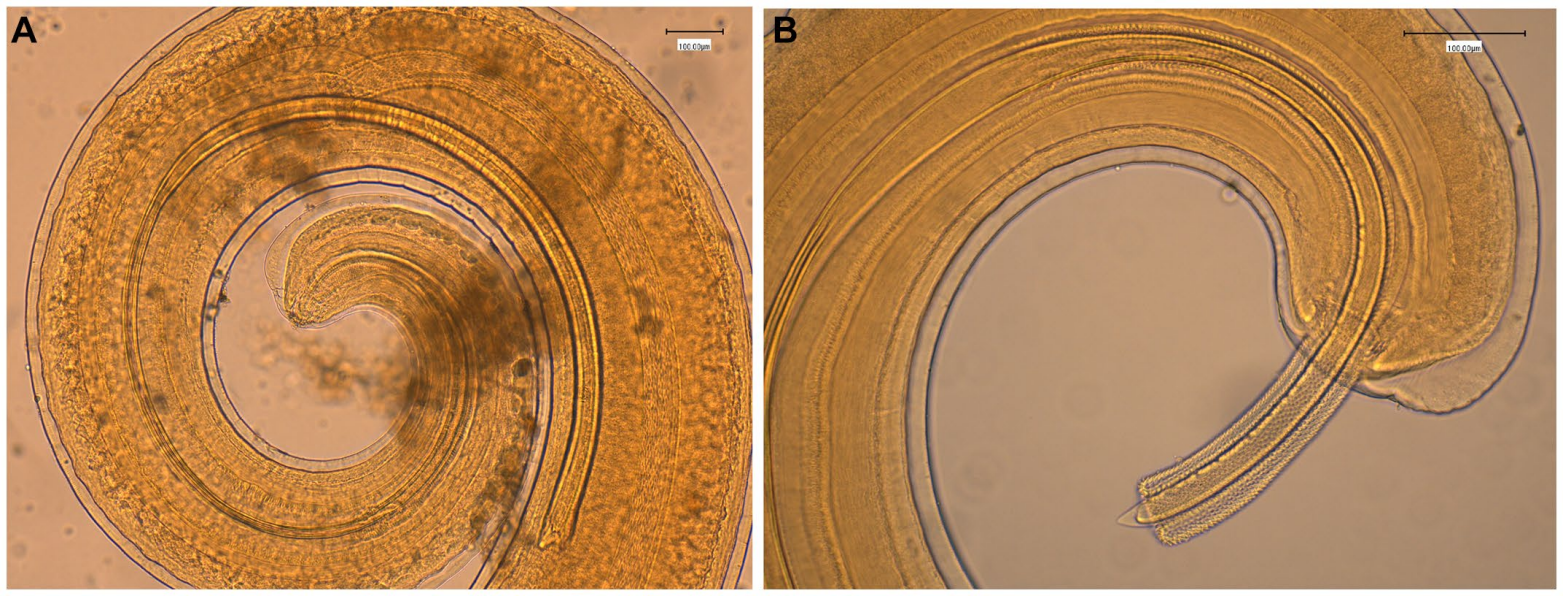
Morphology of male *Trichuris* sp. from *Macaca fascicularis*. **(A)** Posterior end showing the arrowed and invaginated spicule, with distal and proximal cloacal tube and ejaculatory duct. **(B)** Posterior end with evaginated spicule and spicule sheath with spines.

**Figure 3:**
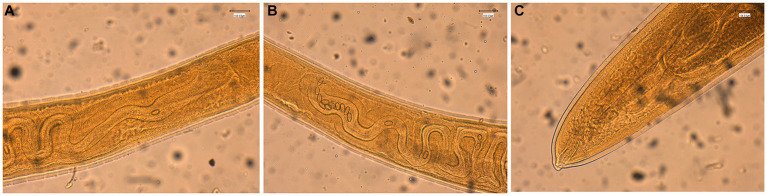
Morphology of female *Trichuris* sp. from *Macaca fascicularis*. **(A)** Vulva region with visible tegument covered by spines. **(B)** Circumvoluted vagina with eggs. **(C)** Posterior end showing the end of uterus and cloaca.

The general gross morphology of *Trichuris* adult specimens collected from *M. fascicularis* and *M. fuscata* intestinal caeca was congruent with a filiform long anterior part and a broad and handle-like posterior part, typical of whipworms. The cuticle presented transversal striation and the anterior portion of the body showed bacillary bands. Males (Figure 2) and females (Figure 3) showed similar morphological features described for *Trichuris trichiura* from *Papio papio* and *M. sylvanus* (31), *Trichuris* sp. from *M. sylvanus* (34, 35) and *T. ursinus* from *Papio ursinus* (36). The eggs measurements ranged from 25.50–27.90 × 54.30–56.80 μm in *Trichuris* from *M. fascicularis* and from 30–35 × 53–61.6 μm in *Trichuris* from *M. fuscata*.

**Figure 4:**
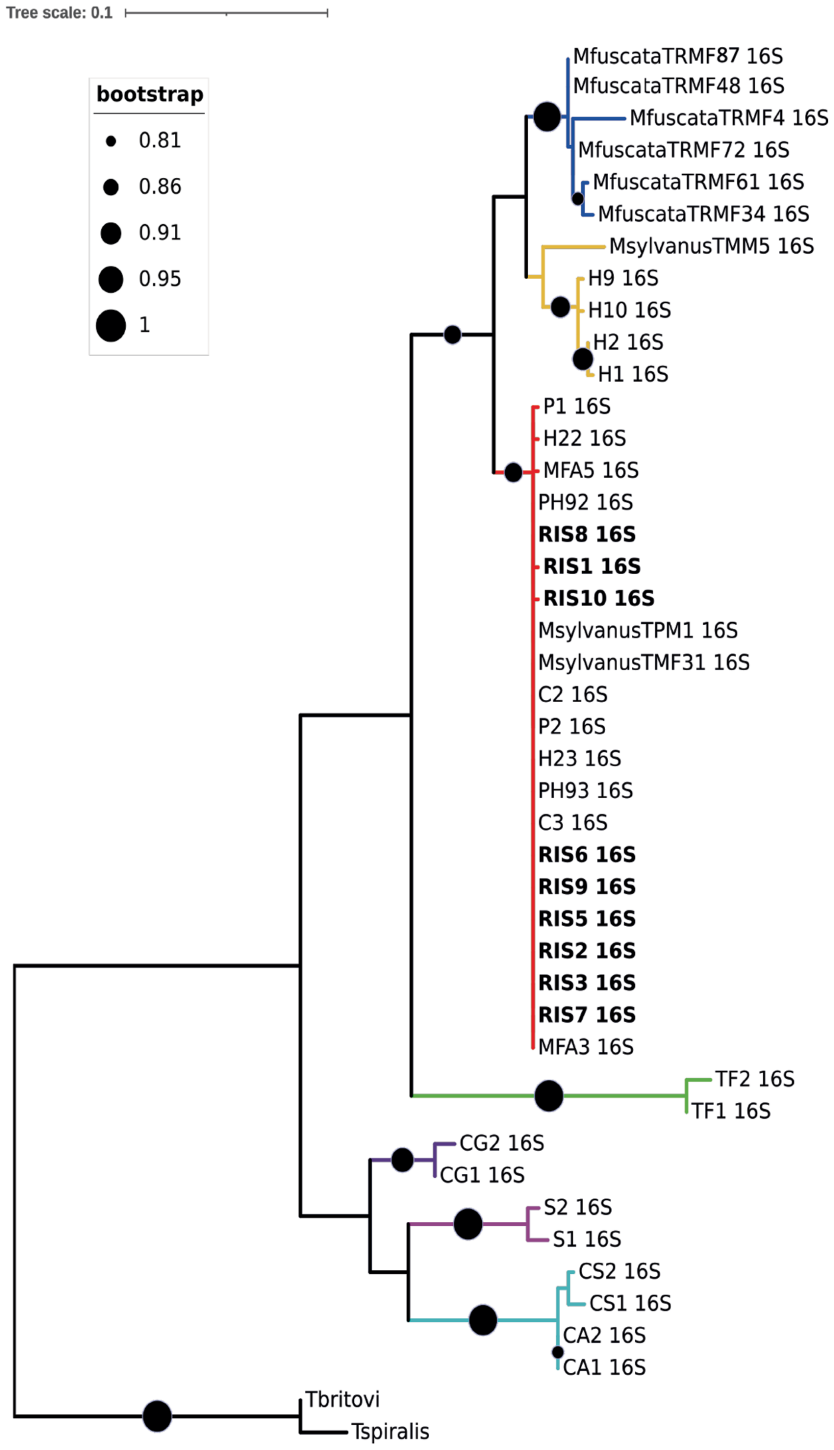
Phylogenetic tree based on *rrn*L of *Trichuris* spp. Maximum likelihood consensus tree of partial mitochondrial *rrn*L of *Trichuris* spp. analyzed in the present study (for specimen codes information see Supplementary File S1). Circles at nodes indicate the bootstrap statistical support, according to the legend on the left. Colors represent clades and sub-clades and correspond to those reported by Rivero et al. (31).

Regarding the molecular characterization, ten high quality *rrn*L sequences (nine from *M. fascicularis* and one from *M. fuscata*) and four *cox*1 sequences (all from *M. fascicularis*) were obtained from the collected nematodes and used for phylogenetic inferences in comparison to GenBank retrieved data, with final datasets of 43 input and 460 bp and of 32 input and 341 bp, respectively. Both phylogenetic trees identified the presence of two main clades, namely “Clade 1” and “Clade 2” (31). The *rrn*L ML consensus tree in Figure 4 described Clade 1 named as the *T. suis* clade, including *Trichuris colobae* as a sister clade of *T. suis* + *Trichuris* sp. from *Chlorocebus*. The *Trichuris* specimens from *M. fascicularis* here analyzed clustered into the “subclade c” of the “Clade 2” or *T. trichiura* clade branch (indicated in red color) (31), with high statistical support (99–100%). The “subclade c” branch included *T. trichiura* individuals collected in a broad host range for primates, such as the Japanese macaque, the Barbary macaque, the green monkey, the baboon, and humans from Africa and Europe. The specimen from *M. fuscata* here collected grouped in the subclade defined as MF in previous reports (branch indicated in blue color) from the same host species living in the Bioparco Zoological Garden of Rome (26).

A similar topology was obtained for the *cox*1 ML consensus tree (Additional file 3), in which specimens of *Trichuris* from *M. fascicularis* were included in the “Clade 2 subclade c” together with *Trichuris* from other macaques and baboons. Such evidences confirmed that specimens infecting *M. fascicularis* here analyzed can be identified as *T. trichiura,* given the similarity with this taxon reported also in other primates, including humans. No good quality sequences were obtained at this marker for *Trichuris* infecting *M. fuscata*.

## 4 Discussion

The present study investigated the presence of intestinal parasites infecting NHP species living in captivity in Italy. Based on morphological analyses from fecal samples of NHPs living at Piano dell’Abatino Park, nine parasite taxa were identified, all of them presenting direct life cycles. However, due to the sampling from premises with multiple hosts, without tracing primate individuals during defecation, the precise estimation of epizootiological parameters such as prevalence, intensity and abundance was not possible.

In this study, *Balantidium/Buxtonella* sp., *E. coli,* and *I. bütschlii* were the most frequently detected parasites. Most parasite taxa identified in this study have been previously reported in captive NHPs in Europe, as is the case of *Trichuris* sp., *Oesophagostomum* sp., *Balantidium* sp., *Giardia* sp., *E. coli* and *I. bütschlii* (19, 22). *Giardia* sp. was found in only one individual, and taking into account that this parasite is usually more frequently found in studies on NHPs in zoos, we should consider that in this case it may not be a true infection but cysts accidentally ingested from the environment. Additionally, due to the intermittent shedding of cysts, in some cases it is necessary the examination of fecal samples on consecutive days (47), and in this study no sampling on consecutive days was performed.

*Balantidium/Buxtonella* sp. was found infecting five of the seven NHP species sampled. Pigs are the main reservoir host of *Balantidium*, while rodents and NHPs may function as alternative reservoir hosts (37). Wild boars are also present at the study site, but in a small number and located in a separate facility from the NHPs. Thus, in this case swine are unlikely to participate in the transmission cycle (even if it cannot be definitively ruled out due to handler’s movements, or by the rain/wind that can easily transport the cysts from one facility to another), while wild rodents are very common within the primate enclosures. For future studies it is highly recommended the use of integrative taxonomy accounting for morphological characteristics combined with molecular approach for species identification, as it has been demonstrated how misleading the cyst morphology-based diagnostics of *Balantidium* sp. and *Buxtonella* sp. can be, leading to ambiguity in the epidemiology of these infections (38). In Italy, both *Buxtonella* sp. and *Balantidium* sp. have been reported, for instance *Buxtonella sulcata* infecting cattle in central Italy (39), and *Balantidium coli* infecting swine in the south of the country (40). Given the uncertainty in the taxonomic assignment, we have chosen to indicate this finding as *Balantidium/Buxtonella* sp.

Molecular testing should be also recommended for the optimal identification of *D. fragilis* (41). In our survey, *D. fragilis-*like was found in samples from *M. fascicularis*, and this parasite was recently reported infecting free-ranging *M. fascicularis* in Indonesia (42). Additionally, future molecular studies to determine the species of the strongyliform larvae found infecting *S. apella* and *M. sylvanus* are required, in particular to confirm or exclude the presence of *Strongyloides* sp., a zoonotic parasite of paramount relevance, reported in Italy both in dogs and humans (43). *Capillaria* sp. was found in one sample of a tufted capuchin monkey, however, the molecular approach for species identification gave negative results, probably due to difficulties in the genomic DNA isolation and/or PCR inhibitors. *Capillaria* sp. has been reported infecting different NHP species (44), including capuchin monkeys: *C. capucinus* in Panama (45) and *C. albifrons* in Ecuador (46). However, these reports were based on microscopy, thus, the use of molecular testing is also here suggested for the identification at species level to elucidate the zoonotic potential.

Trematodes, cestodes and acanthocephalans have been previously reported in free-ranging primates (48). Considering that the methods used in this study allow the detection of these parasite taxa, the lack of findings could be related to the different diets and habits of captive individuals compared to free-ranging NHPs.

Given the close phylogenetic relationship between human and NHPs, continuous parasitological surveys on captive primates should be encouraged for the monitoring of zoonotically transmitted parasites, for instance within conservation and management of threatened primate species, and in the recovery of traded NHPs. In the present study, four out of the seven NHP species under investigation are considered endangered (EN) or vulnerable (VU) by the IUCN, and while no animals hosted at Piano dell’Abatino Park showed clinical signs or symptoms of gastrointestinal origin, two animals died at the Bioparco Zoological Garden of Rome and in the CREMWA, probably because of *Trichuris* infection. So far, *Trichuris* spp. have been reported by morphological and/or molecular characterization in the following *Macaca* species: the Japanese macaque (26, 27, 32), the Barbary macaque (34, 35) and the long tailed macaque (22), the latter investigated only in terms of eggs presence in stool samples without any molecular characterization. Such studies revealed the presence of two separated taxonomic entities able to infect Japanese macaques living in confined environments, one specific to this host, and one shared also with other primates (26, 27, 32). Analogous molecular results were obtained also regarding the Barbary macaque hosted in the Castellar Zoo (Spain), infected by two genotypes within the *T. trichiura* lineage, supported also by morphological data (35).

Here we provide for the first time morphological and molecular data of *T. trichiura* infecting *M. fascicularis,* to share with the scientific community for comparative purposes. We obtained reliable data from the analyses of adult *Trichuris* infecting the dead long tailed macaques hosted at the CREMWA, and despite no molecular data were obtained from fecal samples from the animals hosted at Piano dell’Abatino Park, the eggs size observed in the two sample sites were overlapping, suggesting *T. trichiura* circulation. The long-tailed macaque from the CREMWA analyzed in the present study lived in a colony of around 30 individuals (49), thus the finding of *Trichuris* infection may represent a high risk for the other macaques belonging to the colony. It is also a concern, taking into account that *M. fascicularis* has been recently listed as an endangered species with a decreasing population trend, according to the International Union for Conservation of Nature (IUCN) (50), mainly due to the high demand in the national and international trade, and the hunting for subsistence. Moreover, there is a risk for handlers and visitors in terms of zoonotic transmission. Therefore, it is necessary the constant monitoring to trace the presence of eventual parasitic species of zoonotic interest, in both confined environments and in native areas where NHPs live near or in close contact with humans.

In conclusion, this parasitological survey revealed the presence of potentially zoonotic parasites circulating in NHPs in Italy, providing useful information for the formulation of their management and care plans, and for the elaboration of safety measures for visitors and animal keepers. Regular parasitological surveys in captive NHPs using both microscopy and molecular analyses should be recommended, in order to monitor the impact of parasitosis on the health status of captive NHPs and to properly assess the potential zoonotic transmission risk.

## Data availability statement

The datasets presented in this study can be found in online repositories. The names of the repository/repositories and accession number(s) can be found in the article/Supplementary material.

## Ethics statement

Ethical approval was not required for the study involving animals in accordance with the local legislation and institutional requirements because Biological material taken from alive animals was not invasively collected, while material taken during necropsies was authorized by the ethical approval of the Istituto Zooprofilattico Sperimentale Lazio e Toscana.

## Author contributions

SR: Conceptualization, Funding acquisition, Methodology, Writing – original draft. SC: Conceptualization, Formal analysis, Methodology, Writing – original draft. MMDF: Formal analysis, Writing – review & editing. CD: Resources, Writing – review & editing. FB: Supervision, Writing – review & editing. NC: Resources, Writing – review & editing. SD’A: Conceptualization, Writing – original draft, Writing – review & editing.

## Supplementary material

Supplementary Material 1 *Trichuris* sp. material used for phylogenetic inference based on the partial mitochondrial ribosomal *rrn*L region. Information on specimen codes, host species, GenBank accession number and literature references are provided.

Supplementary Material 2 *Trichuris* sp. material used for phylogenetic inference based on the partial mitochondrial *cox*1 region. Information on specimen codes, host species, GenBank accession number and literature references are provided.

Supplementary Material 3 Maximum likelihood consensus tree of the *Trichuris* spp. partial mitochondrial *cox*1 sequences analyzed in the present study. Numbers at nodes indicate the bootstrap statistical support (for specimen codes information see Additional file 2).
