# Supplementary material for: Intestinal parasites infecting captive non-human primates in Italy
Source: Front Vet Sci. 2024 Jan 8;10:1270202. doi: 10.3389/fvets.2023.1270202 (PMC10804609; doi:10.3389/fvets.2023.1270202)
Supplement: Supplementary Material 1 — Trichuris sp. material used for phylogenetic inference based on the partial mitochondrial ribosomal rrnL region. Information on specimen codes, host species, GenBank accession number and literature references are provided. [file Table_1.DOCX]

Supplementary Material

# Supplementary Material 1

| **Code** | **Host**  **species** | **GenBank**  **accession**  **number** | **Reference** |
| --- | --- | --- | --- |
| TMM5 | *Macaca sylvanus* | MW448471 | Rivero et al. (2021) (44) |
| TRMF4 | *Macaca fuscata* | MW403712 | Cavallero et al. (2021) (24) |
| TRMF34 | *Macaca fuscata* | MW403713 | Cavallero et al. (2021) (24) |
| TRM48 | *Macaca fuscata* | MW403714 | Cavallero et al. (2021) (24) |
| TRMF61 | *Macaca fuscata* | MW403715 | Cavallero et al. (2021) (24) |
| TRMF72 | *Macaca fuscata* | MW403716 | Cavallero et al. (2021) (24) |
| MFA3 | *Macaca fuscata* | MN088542 | Cavallero et al. (2019) (29) |
| MFA5 | *Macaca fuscata* | MN088543 | Cavallero et al. (2019) (29) |
| TF1 | *Trachypithecus francoisi* | KC481232 | Liu et al. (2013) (45) |
| TF2 | *Trachypithecus francoisi* | KC481233 | Liu et al. (2013) (45) |
| TPM1 | *Papio papio* | MW448472 | Rivero et al. (2021) (44) |
| TMF31 | *Macaca sylvanus* | MW448470 | Rivero et al. (2021) (44) |
| TRMF87 | *Macaca fuscata* | OQ569540 | Present study |
| RIS1 | *Macaca fascicularis* | OP328320 | Present study |
| RIS2 | *Macaca fascicularis* | OP328321 | Present study |
| RIS3 | *Macaca fascicularis* | OP328322 | Present study |
| RIS5 | *Macaca fascicularis* | OP328323 | Present study |
| RIS6 | *Macaca fascicularis* | OP328324 | Present study |
| RIS7 | *Macaca fascicularis* | OP328325 | Present study |
| RIS8 | *Macaca fascicularis* | OP328326 | Present study |
| RIS9 | *Macaca fascicularis* | OP328327 | Present study |
| RIS10 | *Macaca fascicularis* | OP328328 | Present study |
| H1 | *Homo sapiens* | GU385218 | Liu et al. (2012) (46) |
| H2 | *Homo sapiens* | AM993017 | Liu et al. (2012) (46) |
| H9 | *Homo sapiens* | KP781899 | Meekums et al. (2015) (47) |
| H10 | *Homo sapiens* | KP781900 | Meekums et al. (2015) (47) |
| H22 | *Homo sapiens* | KU524541 | Hawash et al. (2016) (48) |
| H23 | *Homo sapiens* | KU524542 | Hawash et al. (2016) (48) |
| CA1 | *Chlorocebus aethiops* | MN088565 | Cavallero et al. (2019) (29) |
| CA2 | *Chlorocebus aethiops* | MN088566 | Cavallero et al. (2019) (29) |
| CS1 | *Chlorocebus sabaeus* | MN088559 | Cavallero et al. (2019) (29) |
| CS2 | *Chlorocebus sabaeus* | MN088560 | Cavallero et al. (2019) (29) |
| C2 | *Chlorocebus sabaeus* | KU524595 | Hawash et al. (2016) (48) |
| C3 | *Chlorocebus sabaeus* | KU524596 | Hawash et al. (2016) (48) |
| P1 | *Papio* sp. | KU524558 | Hawash et al. (2016) (48) |
| P2 | *Papio* sp. | KU524559 | Hawash et al. (2016) (48) |
| PH92 | *Papio hamadryas* | MN088578 | Cavallero et al. (2019) (29) |
| PH93 | *Papio hamadryas* | MN088579 | Cavallero et al. (2019) (29) |
| S1 | *Sus scrofa* | KP781894 | Hawash et al. (2016) (48) |
| S2 | *Sus scrofa* | KP781895 | Hawash et al. (2016) (48) |
| CG1 | *Colobus guereza* | MN088583 | Cavallero et al. (2019) (29) |
| CG2 | *Colobus guereza* | MN088584 | Cavallero et al. (2019) (29) |

# Supplementary Material 2

| **Code** | **Host species** | **GenBank accession number** | **Reference** |
| --- | --- | --- | --- |
| RIS1 | *Macaca fascicularis* | OP108821 | Present study |
| RIS3 | *Macaca fascicularis* | OP108822 | Present study |
| RIS5 | *Macaca fascicularis* | OP108823 | Present study |
| RIS6 | *Macaca fascicularis* | OP108824 | Present study |
| TMM5_co1 | *Macaca sylvanus* | MW448471 | Rivero et al. (2021) (44) |
| TMF31_co1 | *Macaca sylvanus* | MW448470 | Rivero et al. (2021) (44) |
| TM18_co1 | *Macaca sylvanus* | LR130784 | Cutillas et al. (2020) (unpublished) |
| TPM1_co1 | *Papio papio* | MW448472 | Rivero et al. (2021) (44) |
| H1_co1 | *Homo sapiens* | GU385218 | Liu et al. (2012) (46) |
| H2_co1 | *Homo sapiens* | AP017704 | Kikuchi et al. (2019) (unpublished) |
| H3_co1 | *Homo sapiens* | KT449826 | Hawash et al. (2015) (49) |
| H4_co1 | *Homo sapiens* | JF690962 | Petrasova et al. (2016) (unpublished) |
| C6_co1 | *Colobus* sp. | FR846241 | Callejón et al. (2016) (unpublished) |
| C1_co1 | *Colobus guereza* | HE653116 | Callejón et al. (2013) (50) |
| C2_co1 | *Colobus guereza* | HE653117 | Callejón et al. (2013) (50) |
| Cg1_co1 | *Colobus guereza* | MK762948 | Cavallero et al. (2019) (29) |
| Cg2_co1 | *Colobus guereza* | MK762949 | Cavallero et al. (2019) (29) |
| Ca3_co1 | *Chlorocebus aethiops* | MK762931 | Cavallero et al. (2019) (29) |
| Ca4_co1 | *Chlorocebus aethiops* | MK762932 | Cavallero et al. (2019) (29) |
| Ca14_co1 | *Chlorocebus aethiops* | MK762941 | Cavallero et al. (2019) (29) |
| Ca15_co1 | *Chlorocebus aethiops* | MK762942 | Cavallero et al. (2019) (29) |
| Cs1_co1 | *Chlorocebus sabaeus* | MK762923 | Cavallero et al. (2019) (29) |
| Cs2_co1 | *Chlorocebus sabaeus* | MK762924 | Cavallero et al. (2019) (29) |
| Cs3_co1 | *Chlorocebus sabaeus* | MK762925 | Cavallero et al. (2019) (29) |
| Mfa1_co1 | *Macaca fuscata* | MK762905 | Cavallero et al. (2019) (29) |
| Mfa5_co1 | *Macaca fuscata* | MK762906 | Cavallero et al. (2019) (29) |
| Mfa6_co1 | *Macaca fuscata* | MK762907 | Cavallero et al. (2019) (29) |
| Mfb2_co1 | *Macaca fuscata* | MK762908 | Cavallero et al. (2019) (29) |
| Mfb3_co1 | *Macaca fuscata* | MK762909 | Cavallero et al. (2019) (29) |
| Mfb4_co1 | *Macaca fuscata* | MK762910 | Cavallero et al. (2019) (29) |
| PU1_co1 | *Papio ursinus* | LT627353 | Callejón and Cutillas (2018) (unpublished) |
| Ph1_co1 | *Papio hamadryas* | JF690963 | Petrasova et al. (2016) (unpublished) |
| Ph91_co1 | *Papio hamadryas* | MK762943 | Cavallero et al. (2019) (29) |
| Ph92_co1 | *Papio hamadryas* | MK762944 | Cavallero et al. (2019) (29) |
