# Supplementary figures and images for: Intestinal parasites infecting captive non-human primates in Italy
Source: Front Vet Sci. 2024 Jan 8;10:1270202. doi: 10.3389/fvets.2023.1270202 (PMC10804609; doi:10.3389/fvets.2023.1270202)

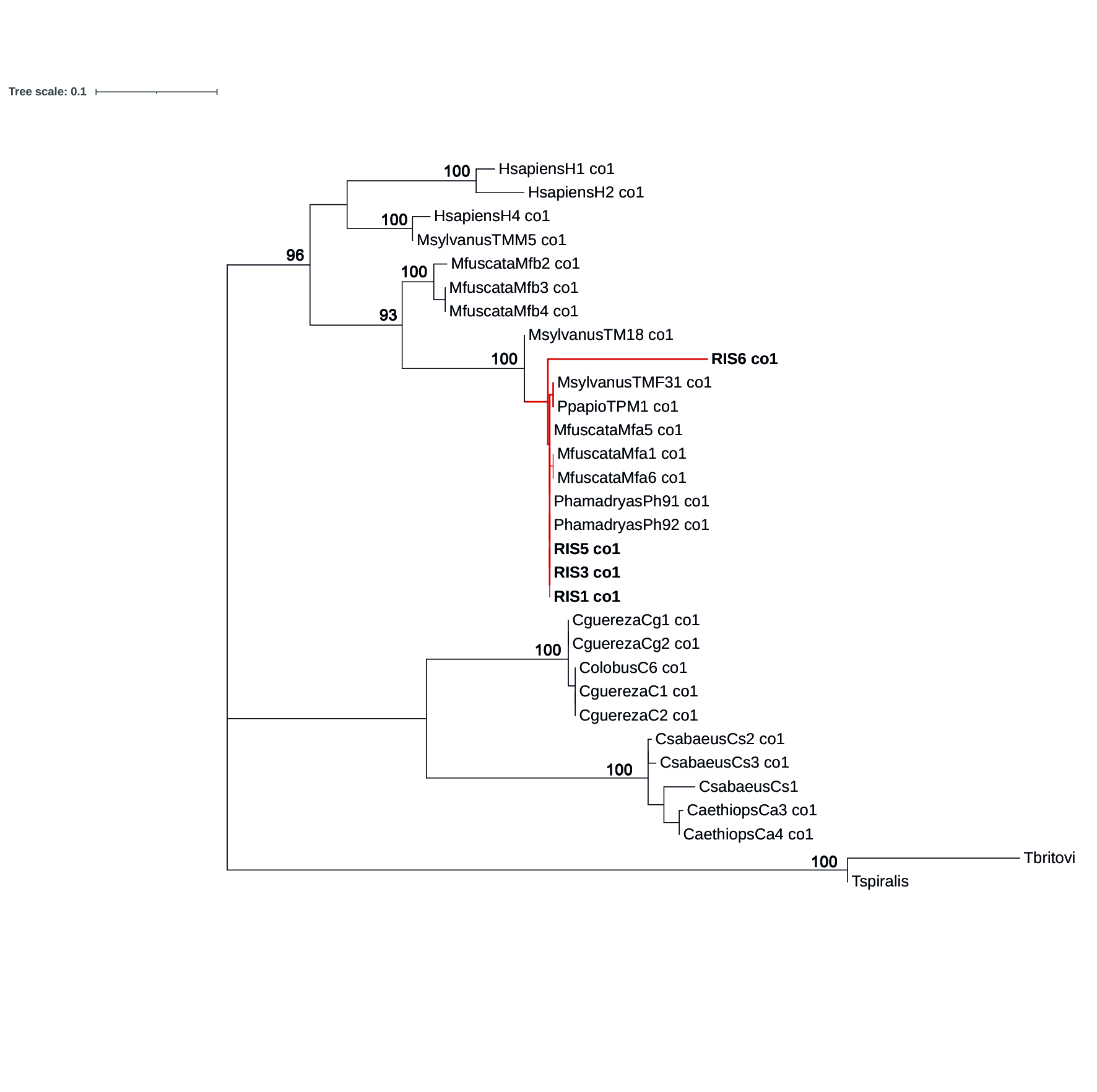

Supplement: Supplementary Material 3 — Maximum likelihood consensus tree of the Trichuris spp. partial mitochondrial cox1 sequences analyzed in the present study. Numbers at nodes indicate the bootstrap statistical support (for specimen codes information see Additional file 2). [file Image_1.TIF]
